# Presence of Calcium Lowers the Expansion of *Bacillus subtilis* Colony Biofilms

**DOI:** 10.3390/microorganisms5010007

**Published:** 2017-02-16

**Authors:** Eisha Mhatre, Anandaroopan Sundaram, Theresa Hölscher, Mike Mühlstädt, Jörg Bossert, Ákos T. Kovács

**Affiliations:** 1 Terrestrial Biofilms Group, Institute of Microbiology, Friedrich Schiller University Jena, Jena 07743, Germany; eisha.r.mhatre@gmail.com (E.M.); sundar.sabitha@gmail.com (A.S.); thoelscher@ice.mpg.de (T.H.); 2 Otto Schott Institute of Materials Research, Friedrich Schiller University Jena, Jena 07743, Germany; mike.muehlstaedt@uni-jena.de (M.M.); Joerg.Bossert@uni-jena.de (J.B.)

**Keywords:** *Bacillus subtilis*, biofilm, calcium, surfactin, sliding, colony expansion

## Abstract

Robust colony formation by *Bacillus subtilis* is recognized as one of the sessile, multicellular lifestyles of this bacterium. Numerous pathways and genes are responsible for the architecturally complex colony structure development. Cells in the biofilm colony secrete extracellular polysaccharides (EPS) and protein components (TasA and the hydrophobin BslA) that hold them together and provide a protective hydrophobic shield. Cells also secrete surfactin with antimicrobial as well as surface tension reducing properties that aid cells to colonize the solid surface. Depending on the environmental conditions, these secreted components of the colony biofilm can also promote the flagellum-independent surface spreading of *B. subtilis*, called sliding. In this study, we emphasize the influence of Ca^2+^ in the medium on colony expansion of *B. subtilis*. Interestingly, the availability of Ca^2+^ has no major impact on the induction of complex colony morphology. However, in the absence of this divalent ion, peripheral cells of the colony expand radially at later stages of development, causing colony size to increase. We demonstrate that the secreted extracellular compounds, EPS, BslA, and surfactin facilitate colony expansion after biofilm maturation. We propose that Ca^2+^ hinders biofilm colony expansion by modifying the amphiphilic properties of surfactin.

## 1. Introduction

Bacteria tend to form sessile, multicellular communities under environmental settings, known as biofilms. In these communities, cells embed themselves in secreted substances that facilitate adherence to surfaces as well as to neighbouring cells. The structures of architecturally complex colonies have been correlated to the general ability of bacteria to develop biofilms [1,2]. When establishing a biofilm, cells of the Gram-positive soil dwelling microbe *Bacillus subtilis* secrete extracellular polysaccharides (EPS), a matrix protein component (TasA), and a hydrophobin protein that assembles on the surface (BslA) [3,4,5,6]. In addition, antimicrobial compounds, including surfactin, are secreted that increase the competitiveness of *B. subtilis* against other microbes [7]. The biofilm matrix components carry out numerous functions in addition to the attachment and the colony structure complexity [8], such as protection from environmental attacks [9], colony spreading [10], or sliding [11,12]. Importantly, colonies lacking EPS and TasA production have reduced morphologies and appear smooth [3]. Cells devoid of BslA lose their hydrophobicity and are prone to water-soluble antimicrobials [4,5]. These above described components, EPS, BslA, and surfactin seem to collectively aid flagellum-independent surface spreading, a coordinated behaviour observed in bacteria [11,12,13].

The expression and synthesis of these secreted products that facilitate biofilm formation and surface spreading are tightly regulated at the level of transcription and affected by various histidine kinases and subsequent cytoplasmic response regulators [6,14,15]. The cytoplasmic and membrane bound histidine kinases (KinA, KinB, KinC, and KinD), in response to dynamic and challenging environmental cues, initiate the phosphorylation of Spo0A (Spo0A~P), the main regulator of various stationary stage processes, via a phosphorelay. The gradual increase in Spo0A~P level influences the cells’ commitment towards certain differentiation processes. KinA and KinB activation results in a large pool of Spo0A~P, sufficient for the cells to undergo sporulation [16,17]. Moreover, KinC and KinD were described to respond to a plethora of signals to maintain a low amount of Spo0A~P that is sufficient to activate the expression of genes responsible for biofilm matrix production [6,15]. Recently, it was demonstrated that KinB and KinC collectively induce *B. subtilis* sliding in a spatiotemporal manner [11]. Apart from being a collective behaviour strategy, sliding is also studied in the context of cooperative strategies in bacteria. Heterogeneity in expression of genes required for the secreted components that aid sliding creates a division of labour between surfactin- and matrix-producing cells at the expanding front of the colony [12].

Examination of the factors and processes that influence colony growth and spreading properties in bacteria facilitate our understanding of bacterial population level behaviours. Here, we report that the presence of Ca^2+^ ions in the environment restricts colony expansion following colony biofilm development. The mature colony formation of *B. subtilis* under laboratory conditions requires three to four days after which the colonies are rugose, structurally complex, and display white chalky patterns attributed to sporulation [1,15]. After maturation of *B. subtilis* biofilms, cells in the middle grow slowly, are encapsulated and well protected, while the peripheral cells continue to grow in the direction of new nutrient sources [18]. Our experiments show that when the growth medium was lacking Ca^2+^ salts, biofilm colonies continue to expand in a way that resembles sliding. Considering that most media used to study biofilm colony structures contain Ca^2+^ salts, this phenomenon is seldom observed. Further, we propose that an interaction between Ca^2+^ and surfactin might be responsible for preventing the colony expansion in the presence of Ca^2+^ in the medium.

## 2. Materials and Methods

### 2.1. Bacterial Strains, Plasmids, and Media

*B. subtilis* DK1042 (naturally competent derivative of the undomesticated NCIB 3610) and its derived mutants were used in this study (Table 1). The strains were inoculated from glycerol cryo-stocks in LB medium (Lysogeny broth, 1% tryptone, 0.5% yeast extract, 0.5% NaCl) overnight before spotting them on the agar plates for complex colony formation. The media used for colony studies are 2×SG [19] and MSgg [1] with 1.5% or 0.7% agar concentration. The original recipes of 2×SG and MSgg contain Ca(NO)_3_ and CaCl_2_, respectively. For generation of strains, genomic or plasmid DNA was transformed into DK1042 using natural competence [20] and the cells were selected on the LB agar with respective antibiotic concentrations. The antibiotic concentrations used were the same as stated previously [15].

For the construction of the P*_bslA_*-*gfp* reporter plasmid (pTB670), the *bslA* promoter region was PCR amplified using primers oTH23 (5′-ACTGAATTCGGGAGCGGGAGGTTCAAGTG-3′) and oTH24 (5′-GCAGCTAGCGCGTTTCATAACAAAATTCC-3’) from *B. subtilis* 3610 genomic DNA, restricted with *Eco*RI and *Nhe*I, cloned into the corresponding sites of p*rrnB*-GFP plasmid [21], and transformed into *Escherichia coli* MC1061.

To construct plasmid pTB497 harbouring a constitutively expressed *gfp* gene, the P_hyperspank_-*gfp* fragment was PCR amplified with primers oTH1 (5′-GCATCTAGAGTTGCTCGCGGGTAAATGTG-3′) and oTH2 (5′-CGAGAATTCATCCAGAAGCCTTGCATATC-3′) from plasmid phy-GFP [22], digested with *Xba*I and *Eco*RI, ligated into plasmid pWK-Sp [23], and transformed into *E. coli* MC1061. Resulting plasmids were verified by sequencing.

### 2.2. Colony Biofilm Formation

For colony spotting, 2×SG or MSgg medium with 1.5% agar were poured and allowed to solidify with closed petri dish lid. Both media were prepared with or without the supplementation of 1 mM Ca(NO_3_)_2_. Once solidified, the plates were opened completely under sterile laminar airflow conditions, and dried for 20 min. Once dried, 2 μL of the overnight grown cultures were spotted on the plate (not more than two colonies per plate), and the lids were closed once the spotted culture dried. The plates were incubated at 30 °C for seven to eight days.

### 2.3. Swarming and Sliding

Swarming and sliding was assayed on LB or 2×SG medium solidified with 0.7% agar. The exact preparation of media and plates were previously described [30]. Plates were incubated at 37 °C and swarming diameter was recorded every hour between 3 and 7 h after inoculation, while sliding was documented after 24 and 48 h.

### 2.4. Imaging and Colony Size Measurements

The colonies grown on the 1.5% agar plates were imaged depending on the medium using an AxioZoom V16 microscope equipped with an AxioCam MRm monochrome camera (Carl Zeiss Microscopy GmbH, Jena, Germany). The colony diameters were also measured to quantitate the colony spread in the presence and absence of the supplemented Ca^2+^. Images were calibrated using Image J version 2.0.0-rc-15. Sliding and swarming disks were recorded using a Nikon D3300 camera (Düsseldorf, Germany) equipped with a Nikon AF-S DX Nikkor 18–55 mm objective.

### 2.5. Growth and Fluorescent Reporter Assays

Overnight cultures of *B. subtilis* strains were diluted 100-fold in 2×SG medium supplemented with different amounts of Ca(NO_3_)_2_; 200 μL aliquots of the culture were placed in the wells of a 96-well plate and incubated under shaken conditions at 30 °C. Growth and fluorescence intensity were recorded every 15 min using an infinite F200PRO plate reader (TECAN Group Ltd., Männedorf, Switzerland).

### 2.6. Surface Tension Measurements

Wild-type or mutant strains were grown overnight in 20 mL 2×SG medium in 50 mL bottles at 37 °C under well agitated conditions. The cells were removed by centrifugation and the culture supernatant was used. The surface tension was measured according to the Wilhelmy plate method using a tensiometer (DCAT 21, DataPhysics, Filderstadt, Germany) interfaced to a computer using the SCAT-33 software, at room temperature (25 °C) and atmosphere pressure. Briefly, 5–10 mL of the supernatant was added to the vessel. The Wilhelmy plate (platinum-iridium plate) used in this study has a wetted length of 40.20 mm. Before each measurement run, the Wilhelmy plate was rinsed with deionized water and subsequently flamed red-hot with a butylene burner. To detect the supernatant’s surface the Wilhelmy plate was moved towards the supernatant’s surface using a motor speed of 1 mm/s and a detection weight threshold of 8.00 mg. Afterwards, the Wilhelmy plate was immersed 3 mm into the supernatant. The measurement was performed at 5 Hz and stopped after attaining a standard deviation below 0.03 mN/m for 50 consecutive measuring points. To calculate the force from the equivalent mass value obtained by the microbalance, the local gravitational acceleration value (9.81485 m/s^2^) for the Otto-Schott-Institute of Materials Research (Jena, Germany), was used. Ten measurements were recorded for each sample, and the experiment was repeated for three biological samples and performed independently twice. The measurements on the various samples were also performed with increasing concentrations of Ca(NO_3_)_2_ to observe the alteration in liquid surface tension.

## 3. Results

### 3.1. Presence of Ca^2+^ Prevents Cells to Spread Out from Matured Biofilm Colonies

**Figure 1:**
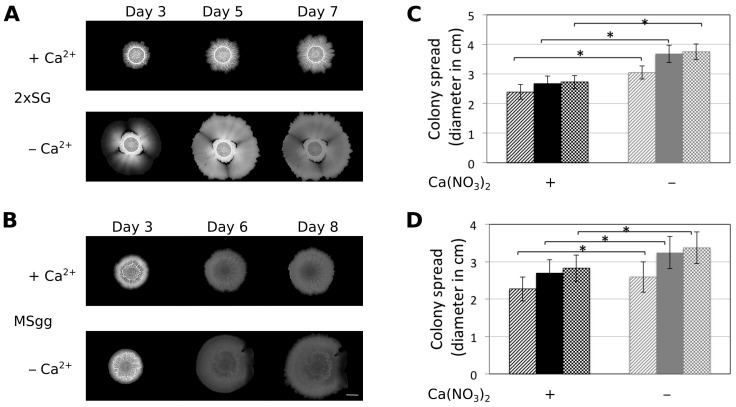
Presence of Ca^2+^ restricts colony expansion. Colonies of *B. subtilis* are shown in the presence and absence of Ca^2+^ on 2×SG (**A**) and MSgg (**B**) media at different days after inoculation. The scale bar at the lower right corner denotes 5 mm. The colony expansion diameters are presented on 2×SG (**C**) and MSgg (**D**) media after three or four (striped), five or six (filled), and seven or eight (checked) days, respectively, after inoculation in the presence (black bars) or absence (grey bars of Ca^2^. The error bars indicate 95% confidence intervals. * denotes significant differences (*p* < 0.05) analysed with paired *t*-test.

When previously examining the impact on Mn^2+^ on colony biofilm development of *B. subtilis* [15], we also tested whether the lack of other components in the medium 2×SG has an effect on the colony biofilm development of various *B. subtilis* strains. Interestingly, we observed that the colonies of *B. subtilis* DK1042 (the naturally competent derivative of the undomesticated NCIB 3610 that forms comparable colony biofilms to NCIB 3610) grown on 2×SG plates without the supplemented Ca(NO_3_)_2_ grew normally until day 3, after which the peripheral cells began to spread and the colony size kept on increasing (Figure 1A). Importantly, no difference in colony growth was observed until three days, and only minor difference was observed in structure. In this paper, we concentrate on the colony size, thus the expansion of the biofilm colonies that denotes the radial expansion of cells after biofilm colony maturation, thus the expansion observed after three days of cultivation. The 2×SG medium contains Ca(NO_3_)_2_ as one of its components. Hence, under normal conditions where all the medium components were supplemented, the colonies were rugose with concentric white chalky patterns around (Figure 1A and [15]). In contrast, when the medium lacked Ca(NO_3_)_2_, the cells at the colony periphery started to expand on the agar surface after three to four days of incubation. To test whether omitting Ca^2+^ or NO_3_^−^ triggers the colony expansion at this later time point of colony development, other salts were tested in 2×SG medium. Neither NO_3_^−^ nor other divalent cations restricted colony expansion similar to Ca^2+^ (Figure S1).

In addition, omitting Ca^2+^ in the biofilm inducing minimal medium, MSgg had a similar impact on the colony spreading (Figure 1B), although the colony biofilm structures differ in the two media. Quantitative measurement of the colony size on 2×SG and MSgg medium revealed that in the absence of Ca^2+^, biofilm colonies spread more and are significantly bigger in size than in the presence of Ca^2+^ (Figure 1C,D). Excluding Ca^2+^ had no major impact on pellicle development on 2×SG medium (Figure S2A).

### 3.2. Ca^2+^ Restricts Flagellum-Independent Expansion of Biofilm Colonies

**Figure 2:**
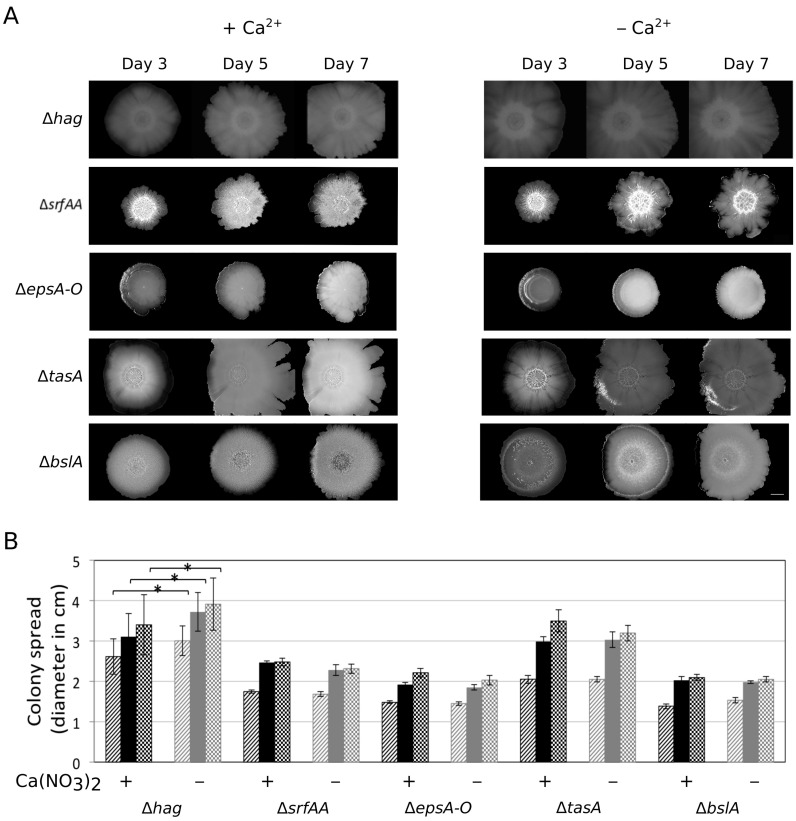
Colony expansion of various mutants of *B. subtilis*. (**A**) The colony images of Δ*hag*, Δ*srfAA*, Δ*epsA-O*, Δ*bslA*, and Δ*tasA* strains are shown three, five, and seven days after inoculation on 2×SG medium in the presence or absence of Ca^2+^. The scale bar indicates 5 mm. (**B**) The colony expansion diameters of the mutants presented in panel A are shown after three (striped), five (filled), and seven (checked) days. Black bars present data in the presence of Ca^2+^, while grey bars indicate the absence of Ca^2^. The error bars indicate 95% confidence intervals. Data was analysed with paired t-test for significantly different samples (* = *p* < 0.05).

The colony expansion (observed after the three days of biofilm development) in the absence of Ca^2+^ was also influenced by nutrient depletion, since cells showed no outgrowth when Ca^2+^ was omitted from 4×SG medium that consisted of twice as much nutrients as 2×SG, while colony expansion was observed when nutrients were reduced (Figure S2B). Dispersal has been described as the ultimate stage of the biofilm lifecycle following nutrient depletion and overcrowding of the sessile population [31]. Colony expansion might be an alternative mechanism to those observed during dispersal. Fleeing from the biofilm is generally facilitated by single cell motility or via small cluster of cells breaking off. As the presence of Ca^2+^ ions restricted the dispersal of complex biofilm colonies, we questioned whether flagellum-dependent motility is necessary for the observed surface spreading. Colony expansion of *B. subtilis* strains lacking the *hag* gene that encoded the flagellin protein was assayed in presence and absence of Ca^2+^. The ∆*hag* strain behaved similar to the *B. subtilis* wild type (WT) as lack of Ca^2+^ supplementation in the medium increased spreading (Figure 2). Interestingly, the spreading of ∆*hag* was more uniform compared to the WT where expansion was observable from small sectors of the matured biofilm colonies (Figure 1).

### 3.3. Importance of the Components Required for Sliding on Colony Expansion

**Figure 3:**
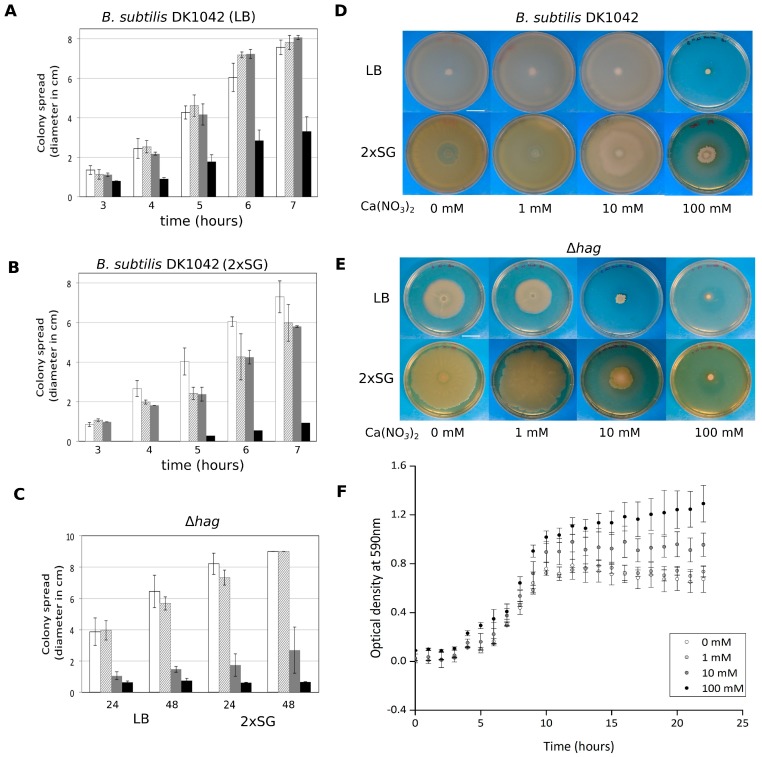
Impact of the presence of Ca^2+^ on swarming and sliding mediated surface colonization of *B. subtilis* DK1042 and ∆*hag* strains, respectively, on Lysogeny broth (LB) and 2×SG medium. Swarming diameter of *B. subtilis* DK1042 strain after 3 to 7 h on LB (**A**) and 2×SG (**B**) media with 0.7% agar without (white bars) or with 1 (stripped bars), 10 (grey bars), 100 mM (black bars) Ca^2+^ supplemented. Sliding diameter of *B. subtilis* ∆*hag* strain (**C**) after 24 and 48 h on LB (left) and 2×SG (right) media supplemented with various amount of Ca(NO_3_)_2_ (labelling similar to S4A). Swarming (**D**) and sliding (**E**) disk of wild type (WT) and ∆*hag* strains, respectively, 24 h after inoculation on LB (above) and 2×SG (below) media with 0.7% agar in the absence or presence of various amounts of Ca^2+^ supplementation. Scale bars indicate 2 cm. Growth properties of *B. subtilis* DK1042 (**F**) in 2×SG medium supplemented with different amount Ca(NO_3_)_2_ from 1 mM to 100 mM.

Surface spreading of *B. subtilis* has been generally examined using semi-solid medium containing 0.5%–0.7% agar. Under these conditions, *B. subtilis* can colonize the agar medium surface using flagellum-dependent swarming or flagellum-independent sliding [11,12,32]. As flagellum-dependent motility was not required for colony expansion, we hypothesized that the observed spreading is similar to sliding that necessitates the collective secretion of EPS, TasA, BslA, and surfactin. Deletion of any of the genes essential for production of these components prevents colony expansion on 2×SG medium without Ca^2+^ supplementation (Figure 2). Therefore, the sliding machinery facilitates the colony expansion after biofilm maturation. A similar trend was observed when the colony sizes of the mutant strains were recorded on MSgg medium in the presence or absence of Ca^2+^ (Figure S3A). To examine if swarming or sliding are influenced by excess Ca^2+^ in the medium, surface colonization of wild-type and ∆*hag* strains of *B. subtilis* exhibiting swarming and sliding, respectively, were assayed on both LB and 2×SG media containing 0.7% agar and different levels of Ca(NO_3_)_2_ (Figure 3). *B. subtilis* swarming diameter was diminished when 100 mM Ca^2+^ was supplemented in both media (Figure 3A,D), while it was somewhat reduced in the presence of 1 and 10 mM Ca^2+^ on 2×SG medium (Figure 3B,D). Moreover, the sliding disk of *B. subtilis* ∆*hag* strain was decreased in the presence of 10 mM Ca^2+^ supplementation in both LB and 2×SG media (Figure 3C,E). These data suggested that Ca^2+^ targeted a component that was required for both swarming and sliding. Importantly, the increased Ca(NO_3_)_2_ concentration had no or minor impact on the growth rate of *B. subtilis* cultivated in liquid 2×SG medium (Figure 3F).

A recent study demonstrated that calcium mineralization in *B. subtilis* colonies impacts biofilm rigidity and scaffolding. This study demonstrated the importance of *lcfA* in bio-mineralization in colonies [33,34]. Incidentally, LcfA is also involved in fatty acid degradation during surfactin production [27]. Nevertheless, mutation in *lcfA* gene did not prevent colony expansion in the absence of Ca^2+^ (Figure S3B).

### 3.4. Colony Expansion on Ca^2+^ Limited Medium Depends on KinB and KinC, the Major Sliding-Inducing Sensor Kinases

**Figure 4:**
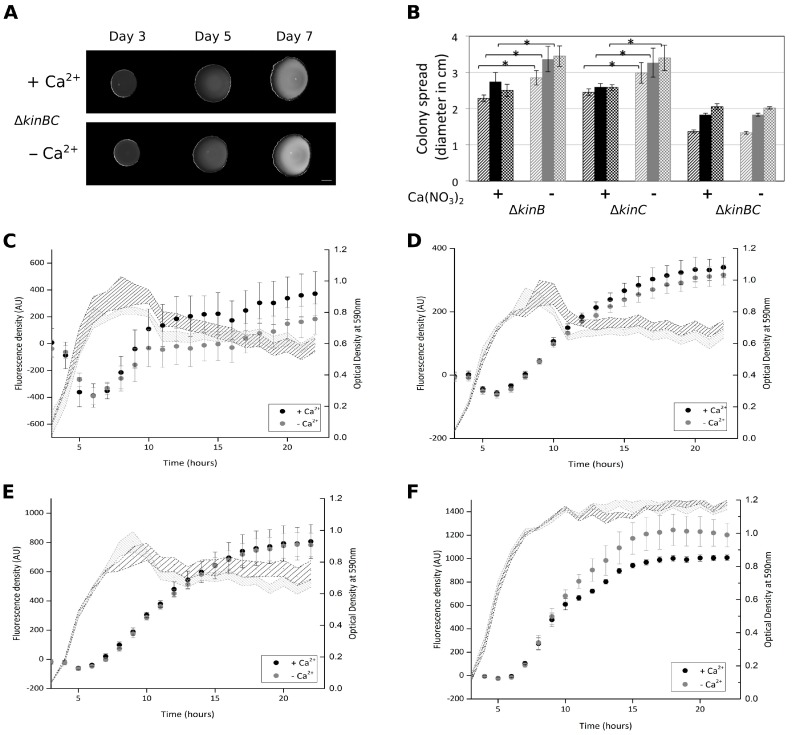
Colony expansion of histidine kinase mutant and expression of selected genes in the presence or absence of Ca^2+^. (**A**) Colony expansion of the Δ*kinB*Δ*kinC* double mutant after three, five, and seven days. Scale bar indicates 5 mm. (**B**) The colony expansion diameters of the Δ*kinB*, Δ*kinC* single, and Δ*kinB*Δ*kinC* double mutant are shown after three (striped), five (filled), and seven (checked) days. Black bars present data in the presence of Ca^2+^, while grey bars indicate the absence of Ca^2+^. The error bars indicate 95% confidence interval. * denotes significant differences (*p* < 0.05) analysed with paired t-test. Relative fluorescence and growth profile (optical density) of *B. subtilis* strains harbouring the P*_srfAA_*-*yfp* (**C**), P*_epsA_*-*gfp* (**D**), P*_tapA_*-*gfp* (**E**), or P*_bslA_*-*gfp* (**F**) constructs in the presence (indicated in black) or the absence (indicated in grey) of Ca^2+^ supplemented in the 2×SG medium.

Since KinB and KinC were reported to activate sliding in *B. subtilis* [11], mutants lacking individual genes coding for the Kin histidine kinases were tested for the colony expansion abilities in the absence of Ca^2+^. None of the single mutants was reduced for colony expansion spreading in Ca^2+^-depleted medium (Figure 4 and Figure S3B). While both KinB and KinC are important for full activation of sliding in *B. subtilis*, only deletion of both kinases results in sliding-deficient phenotype [11]. Consistently, *B. subtilis* harbouring both *kinB* and *kinC* deletions lacked the ability to spread in the absence of Ca^2+^ (Figure 4). As the DegS-DegU two component system was previously described to indirectly activate *bslA* transcription [25,26,35], we tested a strain with a deletion of the *degU* gene for colony expansion. However, the *degU* mutant colony spreading was increased in the absence of Ca^2+^ supplementation (Figure S3B). One explanation for this result could be that although expression of the *bslA* gene is reduced in the *degU* mutant, expression of the *epsA-O* and the *tapA*-*sipW*-*tasA* operons is increased [36,37].

### 3.5. Ca^2+^ Does Not Impact the Expression of the EPS, tasA, and srfA Genes in Planktonic Cultures

The colony expansion in the absence of Ca^2+^ could be related to changes in the expression levels of the *srfAA, epsA-O*, *tasA*, or *bslA* genes. Therefore, the impact of Ca^2+^ supplementation in the 2×SG liquid medium was tested on strain harbouring P*_srfAA_*-*yfp*, P*_epsA_*-*gfp*, P*_tapA_*-*gfp*, or P*_bslA_*-*gfp* fusions. Following the reporter activity over time revealed that the gene expressions of *epsA-O*, and *tapA-sipW-tasA*, and *srfAA-AC* were unaffected, while *bslA* was barely decreased in liquid culture grown in the presence of supplemented Ca^2+^ (Figure 4C–F). Expressions from P*_epsA_*-*gfp* and P*_tapA_*-*gfp* were comparable in colonies in the presence or absence of supplemented Ca^2+^ (data not shown). Importantly, we cannot exclude the possibility that gene expression of *bslA* and *srfA* in matured colony biofilm is increased locally in the absence of Ca^2+^, influencing the expression of genes responsible for colony expansion.

### 3.6. Influence of Ca^2+^ on the Amphiphilic Properties of Surfactin Molecules

**Figure 5:**
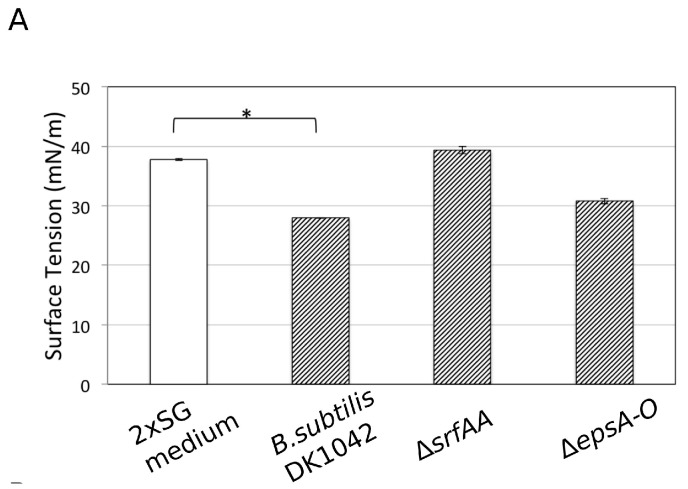

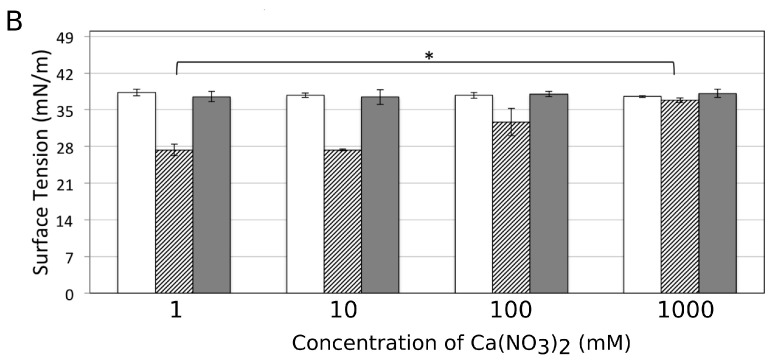
Surface tension measurement of the 2×SG medium and the supernatants of various *B. subtilis* mutants in the presence of different Ca^2+^ levels. (**A**) Surface tension of the 2×SG medium (white bar), wild type, Δ*srfAA*, and Δ*epsA-O* mutant supernatants (black striped bars). (**B**) Surface tension of the 2×SG medium (white bars), the supernatants of WT (striped bars), and of Δ*srfAA* (filled bars) strains in the presence of different Ca^2+^ concentrations. The error bars indicate 95% confidence intervals. Data was analysed with paired t-test for significantly different samples (* = *p* < 0.05).

Next, we addressed the question of how the presence of Ca^2+^ could disturb colony expansion independent of affecting expression of genes related to sliding. Previous studies demonstrated that divalent cations, including Ca^2+^, form complexes with surfactin secreted by *B. subtilis* [38]. Thus, if the Ca^2+^ supplemented in the medium forms a complex with surfactin and alters its amphiphilic property (i.e., surface tension reduction), surfactin facilitated sliding properties might change. To demonstrate that Ca^2+^ can directly influence surfactin properties, surface tensions of spent media (overnight grown culture supernatants) from different strains were recorded in the presence of increasing amounts of Ca^2+^ by the Wilhelmy plate method using a DataPhysics tensiometer DCAT21 [39]. When culture supernatant contained surfactin (e.g., WT and *epsA-O* strain), the liquid surface tension was lower compared to the medium control and the supernatant of the Δ*srfAA* strain (Figure 5A).

The absence or presence of 1 mM of Ca^2+^ had no significant impact on the surface tension of the 2×SG medium. However, when the Ca^2+^ concentration was gradually increased to 100–500 mM, the surface tension values of the medium elevated (Figure 5B). The amount of surplus Ca^2+^ was possibly high enough to form complexes with most of the surfactin molecules in the medium abolishing their surface tension reducing properties. When Ca^2+^ was added to the medium control or the Δ*srfAA* supernatant, the surface tension was not altered and stayed similar to the WT supernatant with high amounts of Ca^2+^.

## 4. Discussion

The quantity of ions in the environment influences various cellular pathways in *B. subtilis*, including biofilm development [15,40,41,42,43]. In our study, we highlighted the role of Ca^2+^ in maintaining the integrity and robust structure of biofilm colonies. In commonly used laboratory media that promote colony biofilm development of *B. subtilis*, cells attach to the agar surface and produce complex robust structures within three to four days. The biofilm matrix components such as EPS, TasA, and BslA play an essential role in colony wrinkleality as well as influence the indentation on the agar surface [44,45]. Interestingly, in the absence of Ca^2+^, peripheral cells in the complex colonies expand radially after four days, likely due to nutrient depletion. In the presence of Ca^2+^, however, the structure is maintained and colony size barely increases. Here, we demonstrated that extracellular polymeric substances and surfactants that are essential for expansion by sliding play an important role in the colony expansion. Mutants that do not produce either surfactin, EPS, the hydrophobin BslA, or the protein component TasA failed to expand from the matured biofilm colonies in medium with reduced Ca^2+^ levels, while the presence or the absence of Ca^2+^ had no major influence on the structural properties of the developing biofilm colonies.

Divalent cations, including Ca^2+^, are known to influence electrostatic interactions and bacterial attachment processes [46,47]. Ca^2+^ is also required for poly-γ-glutamate acid production in *B. subtilis* natto [48]. The influence of Ca^2+^ on surfactin has been extensively studied in X-ray diffraction experiments to demonstrate how the amphiphilic properties of surfactin are reduced during complex formation [38,49]. Moreover, Ca^2+^ also captures and localizes the ionized surfactin molecules in the phospholipid bilayers of the cell membrane. During colony development of *B. subtilis*, Ca^2+^-carbonate present in the agar medium plays an important role during bio-mineralization, establishing scaffold formation and nutrient channelling in the biofilms [33]. The ability of Ca^2+^ to establish complexes with surfactin molecules might explain the lack of colony expansion on an agar medium supplemented with Ca^2+^. Surface tension measurements with bacterial supernatant demonstrated that the high surplus of Ca^2+^ could preclude surfactin dependent reduction of the surface tension. Notably, the amount of Ca^2+^ required for the in vitro inhibition of the surfactin activity was two magnitudes higher than used in the colony experiments. In addition, reduction of sliding and swarming also requires increased Ca^2+^ levels compared to the concentration used for colony biofilms. We hypothesize that this conflicting observation might be resolved by the possibility that the presence of Ca^2+^ ions impact the freshly secreted surfactin at the biofilm colony edge, while Ca^2+^-surfactin complex formation in fluids or in soft agars with increased diffusion is less stable. Colony expansion observed in our experiments on highly viscous medium (i.e., with 1.5% agar) might be more sensitive to alteration in surfactin properties compared to swarming/sliding conditions or liquid medium. Importantly, elevated Ca^2+^ levels in various media were able to reduce swarming and sliding of *B. subtilis*. As both swarming and sliding necessitates the reduction of surface tension by surfactin, these experiments further supported that interaction of Ca^2+^ and surfactin has great impact on surface spreading on soft agar medium.

This study adds to our understanding of rugose colony structure development in *B. subtilis* and the factors involved in maintaining these structures. The presence of Ca^2+^ in the medium not only prevented the expansion of the cells from the colonies but also restricted them in the nutritionally depleted environment, thus probably indirectly influencing late stationary processes such as sporulation. The cells in the biofilm colonies were previously described to form white rugose structures due to sporulation. Thus, Ca^2+^ has a substantial impact on the fate of colonies and the differentiation properties of these complex biofilm populations. In addition, our results might have implications towards surface engineering of various materials related to biofilm formation and bacterial colonization in general.

## Figures and Tables

**Table 1:** Strains used in the study.

Strain	Genotype	Reference, Source, or Construction
DK1042	3610 *comI*^Q12I^	[20]
TB500	3610 *comI*^Q12I^ *amyE*::P_hysperpank_-*gfp*(*spec^R^*)	pTB497 → DK1042
TB602	3610 *comI*^Q12I^ Δ*tasA::spec^R^*	TB163 [11] → DK1042
TB277	3610 *comI*^Q12I^ Δ*srfAA::Cm*	RG551 [11] → DK1042
TB530	3610 *comI*^Q12I^ Δ*hag::neo*	TB24 [11] → TB500
TB524	3610 *comI*^Q12I^ Δ*epsA-O::tet^R^*	DL1032 [24] → TB500
TB526	3610 *comI*^Q12I^ Δ*bslA::cm^R^*	NRS 2097 [25] → TB500
TB398	3610 *comI*^Q12I^ Δ*kinA::mls^R^*	JH12638 [11] → DK1042
TB399	3610 *comI*^Q12I^ Δ*kinB::tet^R^*	JH19980 [11] → DK1042
TB400	3610 *comI*^Q12I^ Δ*kinC::spec^R^*	BAL393 [11] → DK1042
TB401	3610 *comI*^Q12I^ Δ*kinD::cm^R^*	BAL691 [11] → DK1042
TB402	3610 *comI*^Q12I^ Δ*kinE::cm^R^*	BAL692 [11] → DK1042
TB672	3610 *comI*^Q12I^ Δ*kinB::tet^R^* Δ*kinC::spec^R^*	TB400 → TB399
TB656	3610 *comI*^Q12I^ Δ*kinC::spec^R^* Δ*kinD::cm^R^*	TB400 → TB401
TB671	3610 *comI*^Q12I^ Δ*degU::neo^R^*	Δ*degU* [26]→ DK1042
TB51	3610 *comI*^Q12I^ Δ*lcfA::mls^R^*	MW2 [27] → DK1042
TB363	3610 *comI*^Q12I^ *sacA*::P*_epsA_*-*gfp*(*neo^R^*)	[28]
TB373	3610 *comI*^Q12I^ *sacA*::P*_tapA_*-*gfp*(*neo^R^*)	[28]
TB685	3610 *comI*^Q12I^ *amyE*::P*_bslA_*-*gfp*(*cm^R^*)	pTB670 → DK1042
TB740	3610 *comI*^Q12I^ P*_srfAA_*-*gfp*(*spec^R^*)	BD4720 [29] → DK1042

## Supplementary Materials

The following are available online at www.mdpi.com/2076-2607/5/1/7/s1, Figure S1: Ca^2+^ specifically reduces colony expansion, Figure S2: Impact of Ca^2+^ on pellicle formation and colony spreading at different nutrient concentrations, Figure S3: Colony expansion of various strains on MSgg and 2×SG medium.
