# Supplementary material for: Presence of Calcium Lowers the Expansion of Bacillus subtilis Colony Biofilms
Source: Microorganisms. 2017 Feb 16;5(1):7. doi: 10.3390/microorganisms5010007 (PMC5374384; doi:10.3390/microorganisms5010007)
Supplement: Supplementary file 1 [file microorganisms-05-00007-s001.pdf]

Supplementary Material for

Presence of Calcium Lowers the Expansion of Bacillus subtilis Colony Biofilms

Eisha Mhatre [1], Anandaroopan Sundaram [1], Theresa Hölscher [1], Mike Mühlstädt [2], Jörg Bossert [2], Ákos T. Kovács [1,*]

1 Terrestrial Biofilms Group, Institute of Microbiology, Friedrich Schiller University Jena, 07743 Jena, Germany; eisha.r.mhatre@gmail.com (E.M.); sundar.sabitha@gmail.com (A.S.); thoelscher@ice.mpg.de (T.H.),

2 Otto Schott Institute of Materials Research, Friedrich Schiller University Jena, 07743 Jena, Germany; mike.muehlstaedt@uni-jena.de (M.M.); Joerg.Bossert@uni-jena.de (J.B.)

* Correspondence: akos-tibor.kovacs@uni-jena.de

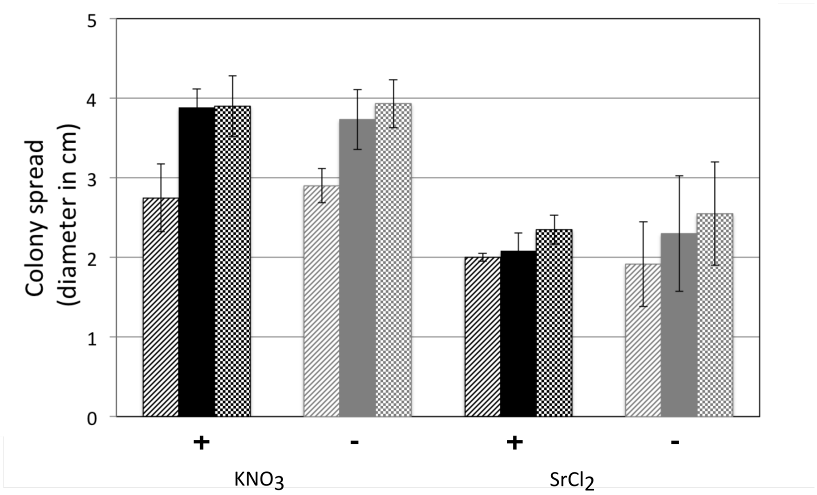

Figure S1. Ca^{2+} specifically reduce colony escape. The colony expansion diameters of *B. subtilis* DK1042 are shown after 3 (striped), 5 (filled), and 7 (checked) days. Black bars present data in presence, while grey bars indicate in absence of KNO_3 and $SrCl_2$ in the 2×SG medium. KNO_3 was used to observe the impact of NO_3^-, while $SrCl_2$ was applied to assay the influence of another divalent cation. The error bars indicate 95% confidence intervals.

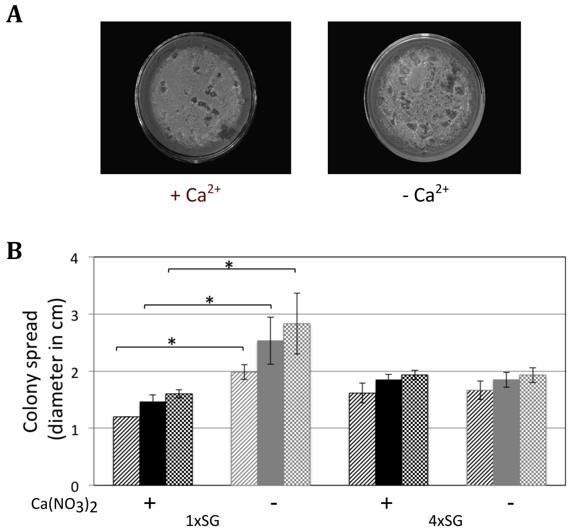

Figure S2. Impact of Ca^{2+} on pellicle formation and colony spreading at different nutrient concentrations. (A) Pellicle formation of *B. subtilis* DK1042 on 2×SG medium in the presence (left) and absence (right) of Ca^{2+} supplementation after 3 days. (B) The colony expansion diameters of the *B. subtilis* DK1042 are shown on 1×SG and 4×SG media with 1.5% agar after 3 (striped), 5 (filled), and 7 (checked) days. The black bars present data in presence, while grey bars indicate in absence of Ca^{2+} supplementation in the media. The error bars indicate 95% confidence intervals. * denotes significant differences ($p<0.05$) analyzed with paired t-test.

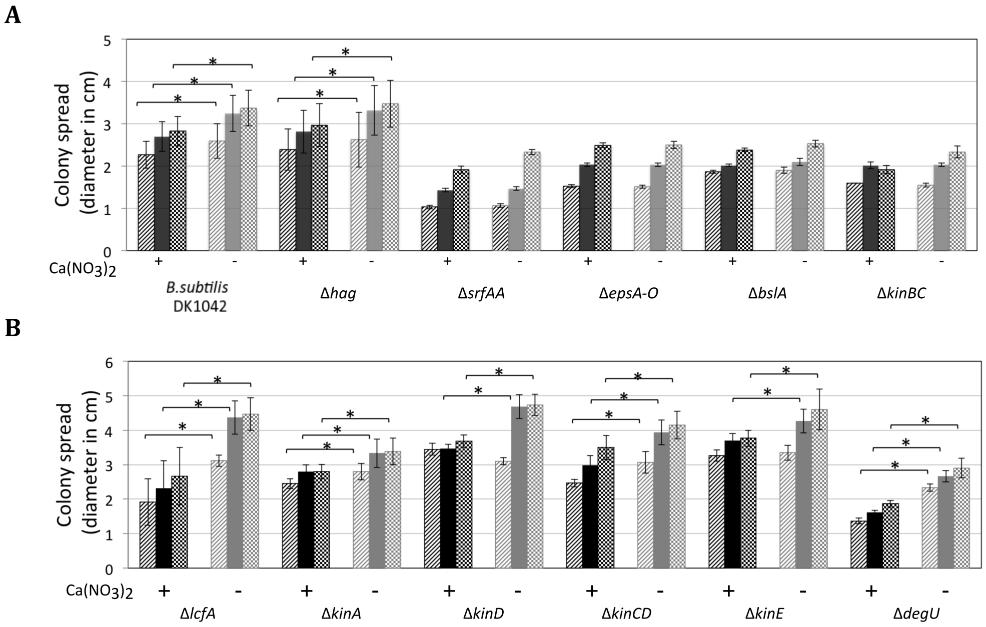

Figure S3. Colony escape of various strains on MSgg (A) and 2×SG (B) medium. (A) The colony expansion diameters of the *B. subtilis* DK1042 and its derivatives, Δ*hag*, Δ*srfAA*, Δ*epsA-O*, Δ*bslA*, and Δ*kinB*Δ*kinC* are shown on MSgg medium after 4 (striped), 6 (filled), and 8 (checked) days. (B) The colony expansion diameters of the *B. subtilis* Δ*lcfA*, Δ*kinA*, Δ*kinB*, Δ*kinC*, Δ*kinD*, Δ*kinE*, Δ*kinC*Δ*kinD*, and Δ*degU* are shown on 2×SG medium after 3 (striped), 5 (filled), and 7 (checked) days. Black bars present data in presence, while grey bars indicate in absence of Ca^{2+} supplementation in the respective media in both panels. The error bars indicate 95% confidence intervals. * denotes significant differences ($p<0.05$) analyzed with paired t-test.
